# Supplementary material for: Proximity does not contribute to activity enhancement in the glucose oxidase–horseradish peroxidase cascade
Source: Nat Commun. 2016 Dec 22;7:13982. doi: 10.1038/ncomms13982 (PMC5196434; doi:10.1038/ncomms13982)
Supplement: Supplementary Information — Supplementary Methods, Supplementary Figures, Supplementary References [file ncomms13982-s1.pdf]

Supplementary Methods

Michaelis-Menten kinetics of glucose oxidase. The initial activity of GOx in various concentrations of glucose was determined by the HRP-coupled colorimetric assay with 1 nM GOx and 20 nM HRP in PBS (pH 7.4). The activity assays were carried out on a UV-Visible spectrophotometer (Evolution 201, Thermo Scientific, US). Briefly, 20 μL of 50 nM GOx was added to 980 μL of substrate solution (in PBS buffer, pH 7.4) to initiate the reaction. The substrate buffer contains 2 mM ABTS, 20 nM HRP and different concentrations of glucose (1 mM to 90 mM). The increase in absorbance at 415 nm was continuously recorded for 2 min and the progress curve was fitted with a linear fit to calculate the GOx activity.

The k_{cat} of GOx and K_m were determined as 250 s^{-1} and 15 mM, respectively. The turnover number is close to the previous study by Fu *et al.*, where the k_{cat} was 310 s^{-1}, but their K_m is 3.5 mM.[1] Therefore the apparent activity of our GOx in 1 mM is 15 nM s^{-1}, but their GOx is 70 nM s^{-1}. For the kinetics of HRP, we measured a k_{cat} of 32.7 s^{-1} and K_m of 2.5 μM. In comparison, Fu *et al.* obtained a k_{cat} of 32 s^{-1} and K_m of 2.3 μM,[2] where the parameters are almost the same. These comparisons indicate that the rate limiting enzyme is GOx in this study, but may be HRP in previous studies by Fu *et al*.

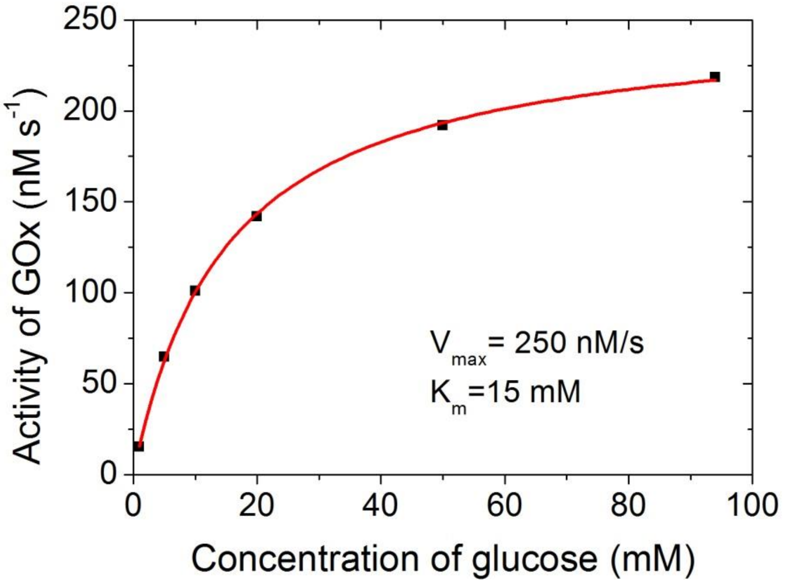

Supplementary Figure 1. The dependence of the activity on the substrate concentration of GOx shows Michaelis-Menten kinetics.

Calibration of H_2O_2 concentration. The concentration of commercial H_2O_2 was determined by HRP-ABTS enzymatic reaction. 20 μL of 1 μM HRP and 2 mM ABTS were added into the H_2O_2 solution at various concentrations (in 10 mM PBS, pH 7.4). The highest absorbance of

ABTS$^{+\bullet}$ at 600 nm was recorded. A typical curve was shown in **Supplementary Figure 2**, and the calibration curve is shown in **Supplementary Figure 3.**

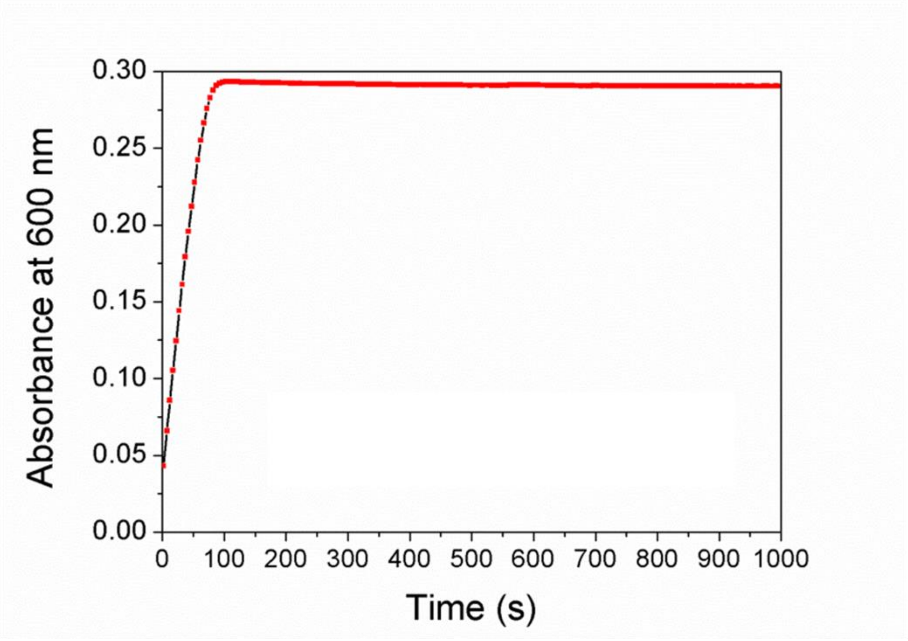

Supplementary Figure 2. Typical curve for determining the actual concentration of H_2O_2. In this figure, the determined concentration of H_2O_2 is 13.3 μM, while the stated concentration is 20 μM (according to the specification).

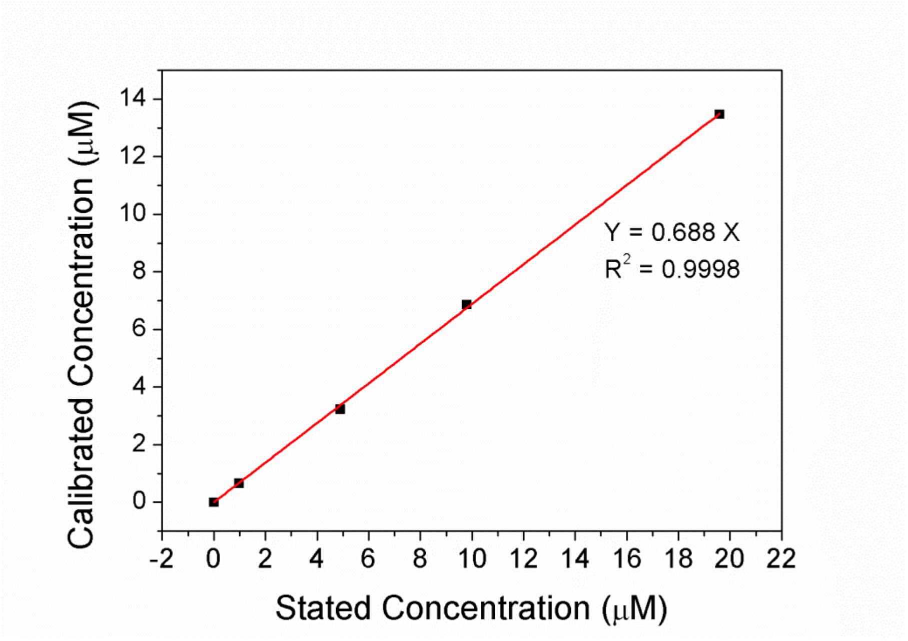

Supplementary Figure 3. Calibration curve of the H_2O_2 concentration.

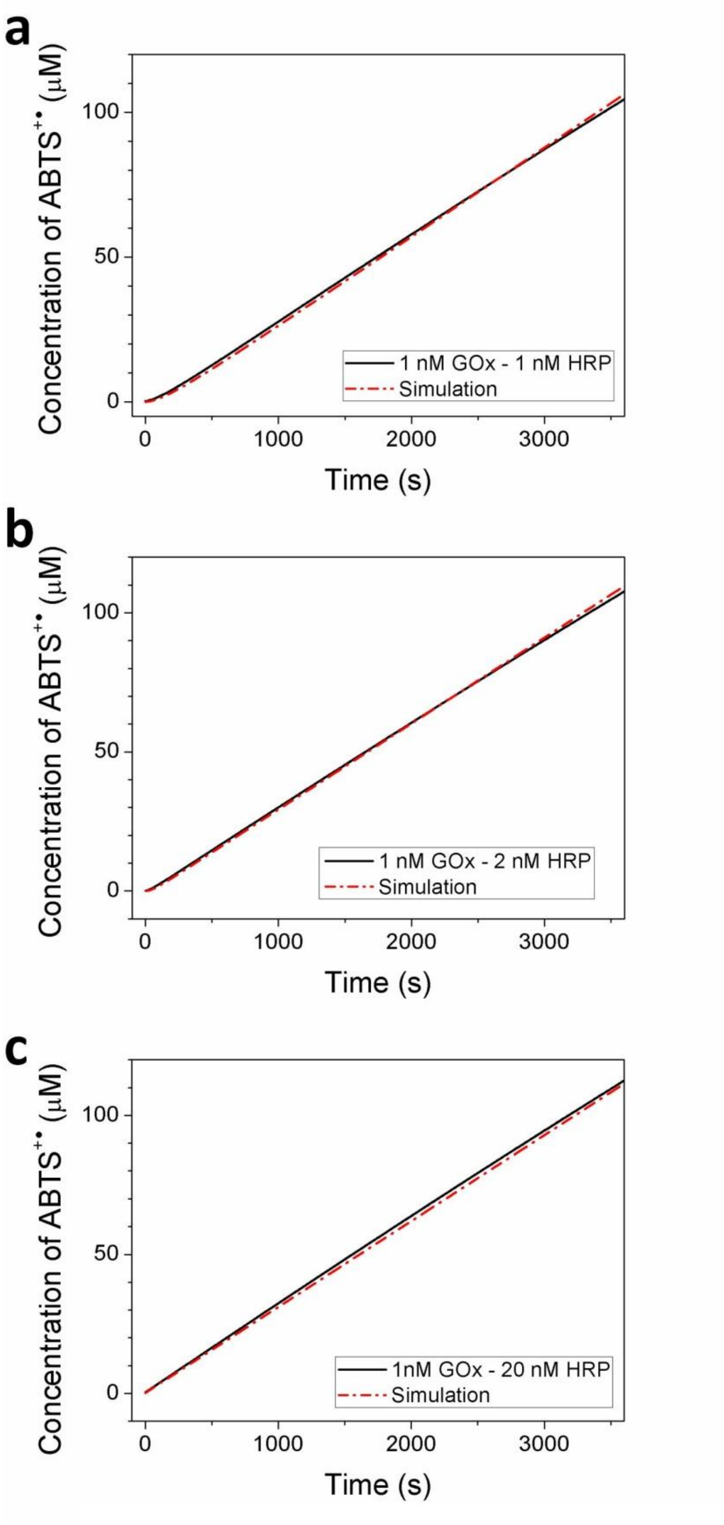

Supplementary Figure 4. Time course curves of $ABTS^{+\bullet}$ production in the presence of 1 nM, 2 nM, 20 nM HRP and the simulation curves.

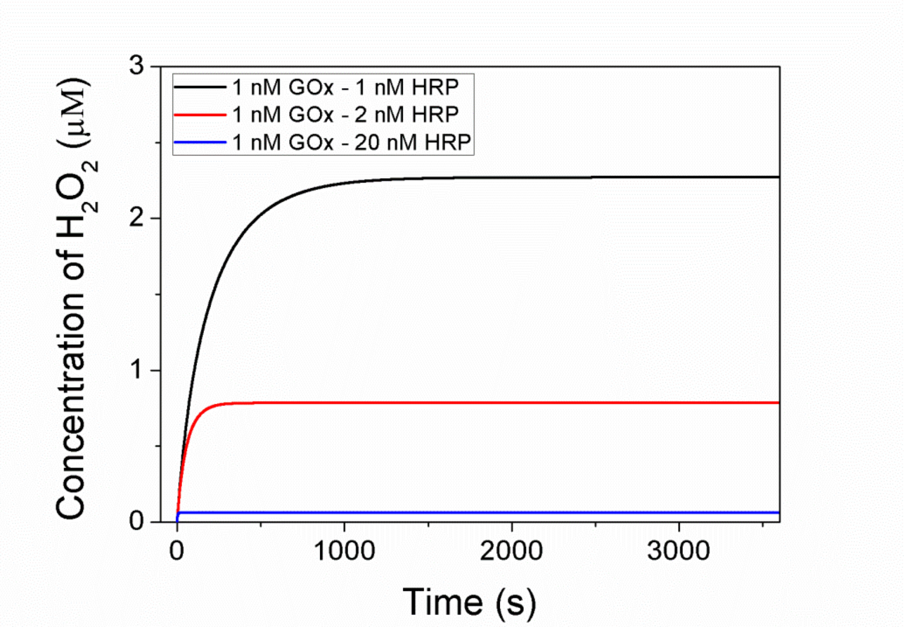

Supplementary Figure 5. The accumulation of H_2O_2 in the bulk solution in the presence of 1 nM GOx and 1 M, 2 nM or 20 nM HRP (based on the model).

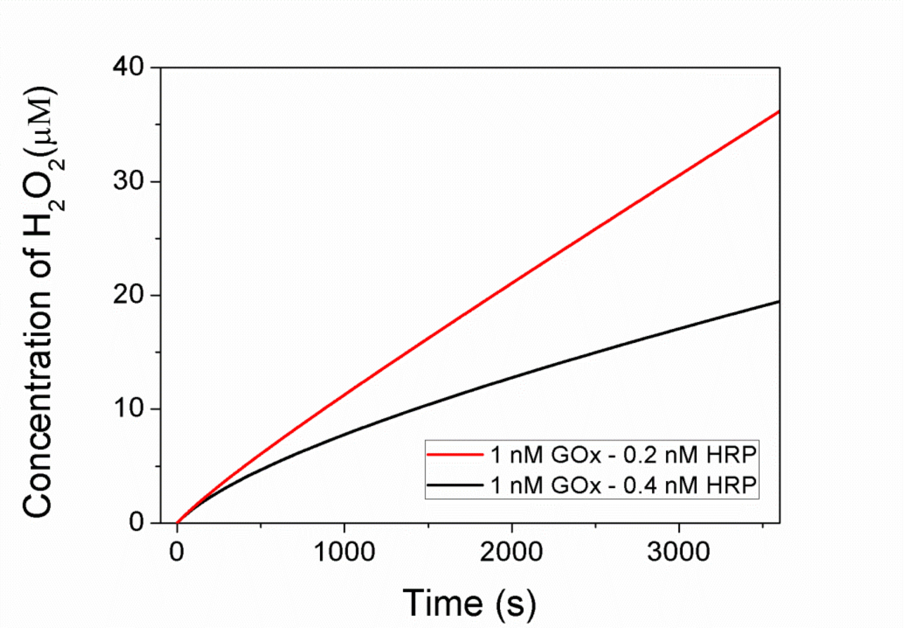

Supplementary Figure 6. The accumulation of H_2O_2 in the bulk solution in the presence of 1 nM GOx and 0.2 nM or 0.4 nM of HRP (based on the model).

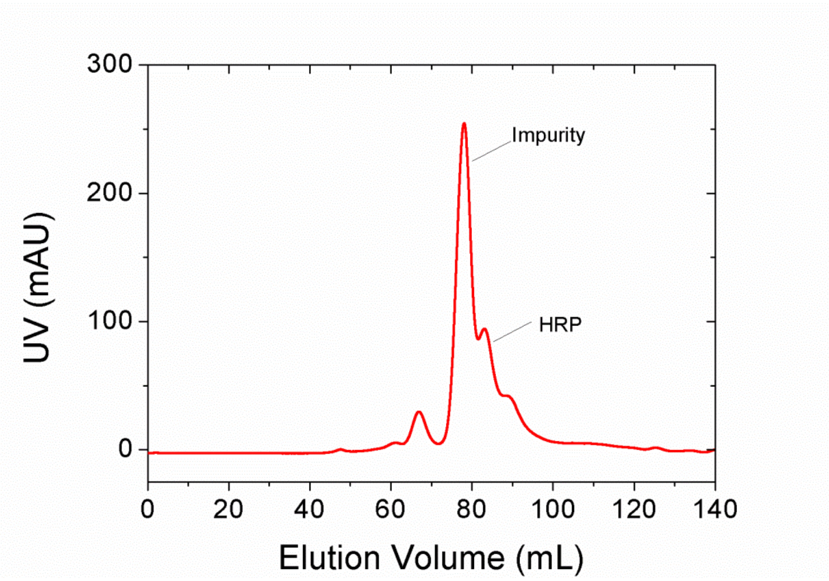

Supplementary Figure 7. Size exclusion chromatograms of HRP before purification.

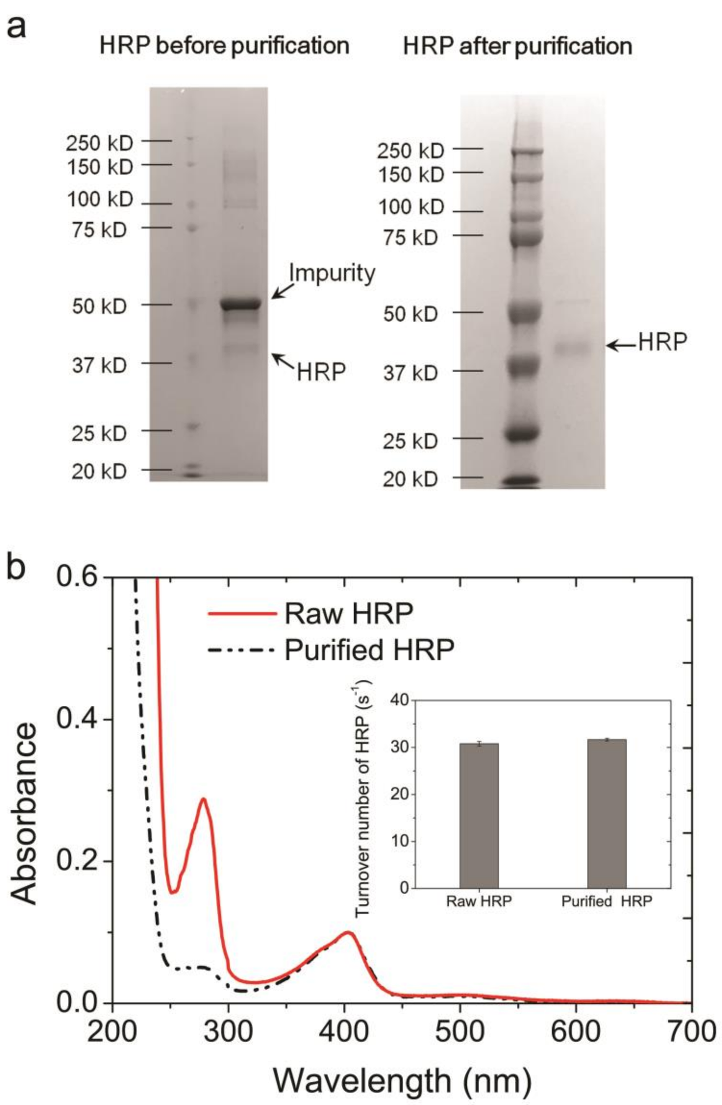

Supplementary Figure 8. a) SDS-PAGE of HRP before and after purification. b) Spectra of the

raw HRP and the purified HRP. The insert indicates the activity of HRP does not significantly changed after purification (the concentrations are determined by the absorbance at 403 nm).

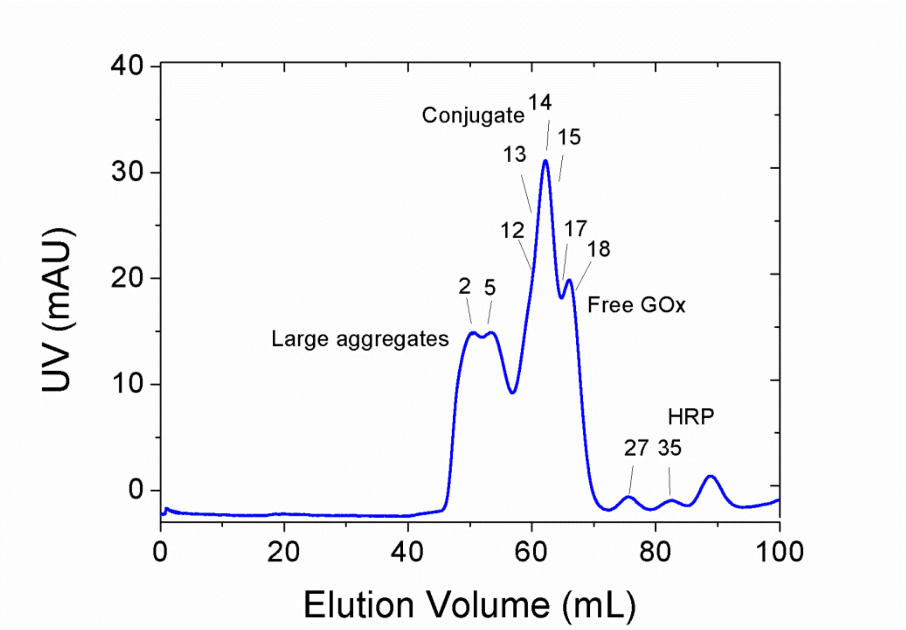

Supplementary Figure 9. Size exclusion chromatograms of HRP-GOx conjugate mixture, the fraction numbers are indicated on the curve.

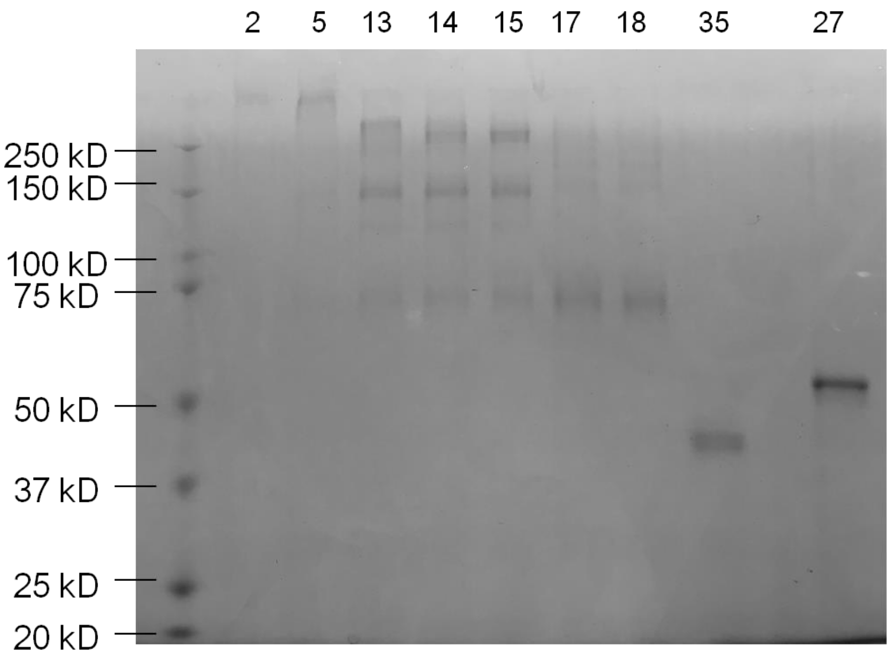

Supplementary Figure 10. SDS-PAGE of fractions from the SEC. Fraction 2 and 5 are the large aggregates of enzymes, fraction 12 to 15 are enzyme conjugates; fraction 17 and 18 are the free GOx, fraction 27 (10-fold concentrated) is the impurity from the raw HRP, fraction 35 (10-fold concentrated) is free HRP.

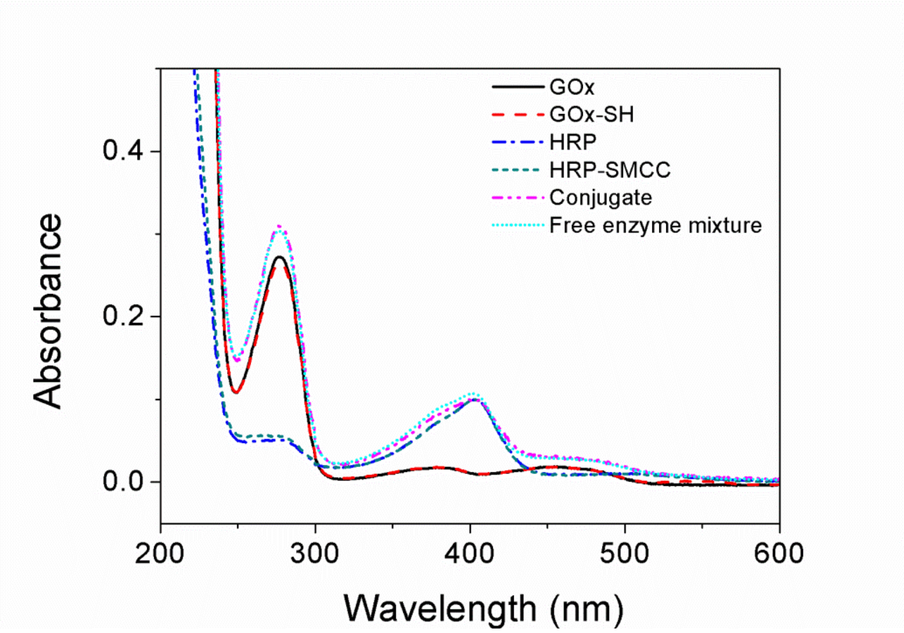

Supplementary Figure 11. Spectra of GOx, HRP, mixture of free GOx and HRP, GOx-SH, HRP-SMCC, and the GOx-HRP conjugate. The concentration of each enzyme solution was adjusted to 1 μM based on the absorbance at 280 nm and 403 nm.

Activity assay of catalase. The activity of catalase was determined by the initial consumption rate of H_2O_2 on a spectrophotometer at 240 nm. The excitation coefficient of H_2O_2 is 43.6 M^{-1} cm^{-1}. The molecular weight of catalase is 250 kDa and the extinction coefficient $E^{1\%}$ = 36.5 (at 276 nm). The activity assay was carried out with 1 nM catalase in PBS, pH 7.4. The kinetics of catalase is shown in **Supplementary Figure 12**.

However, the activity of catalase is significantly inhibited by ABTS. To determine the inhibition effect, we monitored the decomposition process of H_2O_2 in the presence of 1 nM catalase and 2 mM ABTS via HRP-ABTS reaction, as shown in **Supplementary Figure 13**.

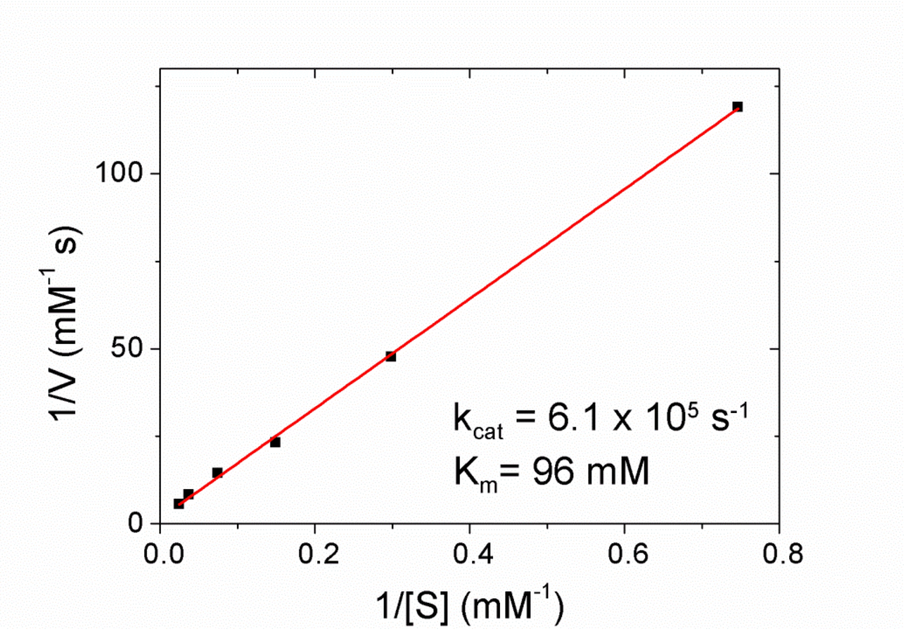

Supplementary Figure 12. Kinetics of catalase.

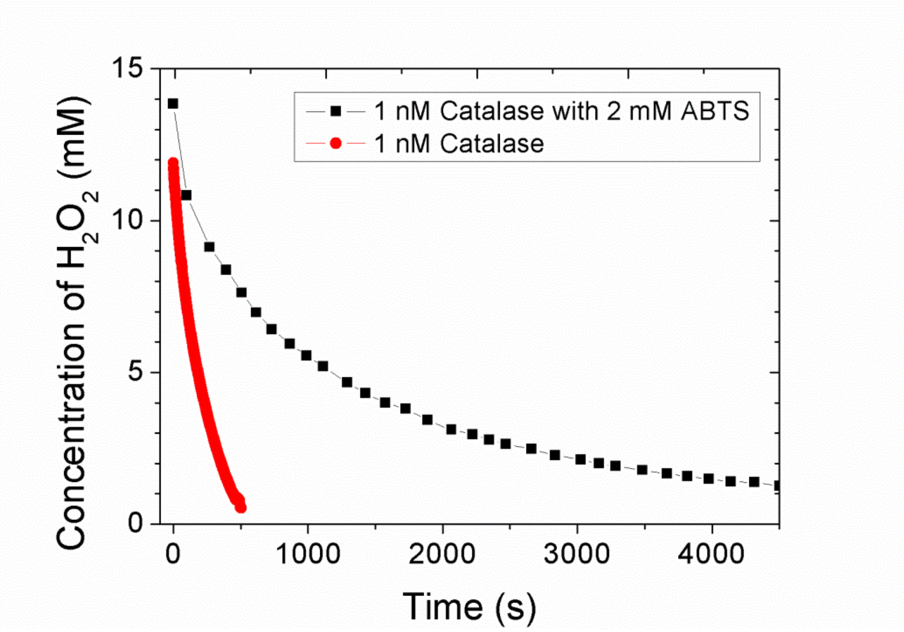

Supplementary Figure 13. Inhibition effect for catalase by ABTS.

Simulation of catalase competing cascade. Since the steady state concentration of H_2O_2 in GOx-HRP cascade is very low (on the order of micromoles, relative to the K_m of catalase), we can simplify the reaction rate of catalase as Supplementary Equation (1):

$$r_{CAT} = k_{app}[E_{CAT}][H_2O_2] \tag{1}$$

Then, the model for the catalase competition experiment is described by Supplementary Equations (2-7):

$$
\begin{cases}
r_1 = k_{cat,1}[E]_1 & (2) \\
r_2 = \dfrac{V_{max,2}[I]_t}{[I]_t + K_{m,2}} & (3) \\
r_{CAT} = k_{app}[E_{CAT}][I]_t & (4) \\
[I]_t = r_1 t - \int_0^t r_2 dt - r_{CAT} t & (5) \\
[P] = 2 \cdot \int_0^t r_2 dt & (6) \\
[I]_{t_0} = 0 & (7)
\end{cases}
$$

From the low concentration range of **Supplementary Figure 13**, we obtained a k_{app} of 6×10^5 $M^{-1}s^{-1}$. We simulated the throughput curve of the cascade competing GOx-HRP cascade. The simulation curves agree well with the experiments (**Supplementary Figure 14**).

The catalase competition with free GOx and HRP reaction is shown in **Supplementary Figure 15.**

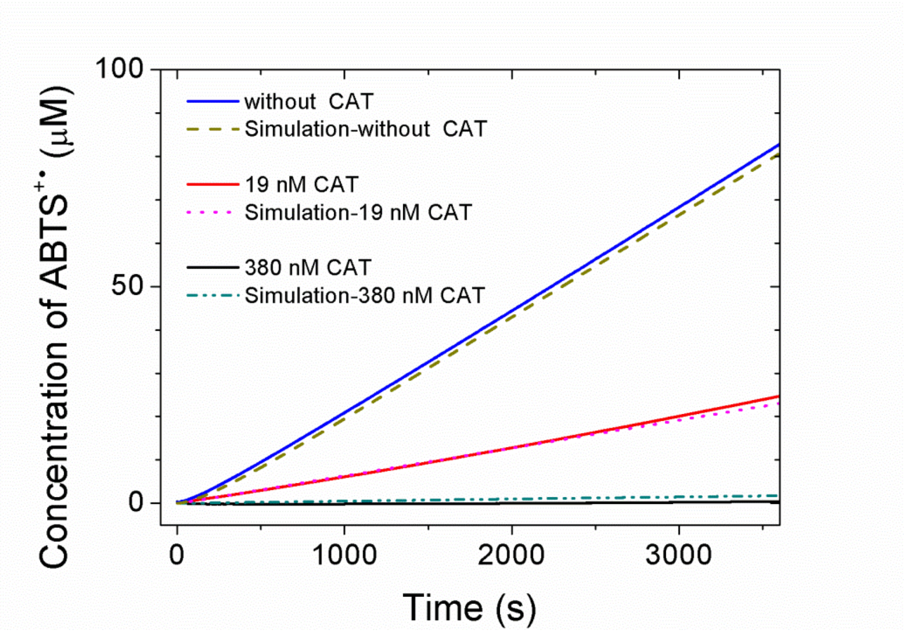

Supplementary Figure 14. Experiment and modeling of cascade reaction of GOx-HRP conjugate with catalase competition.

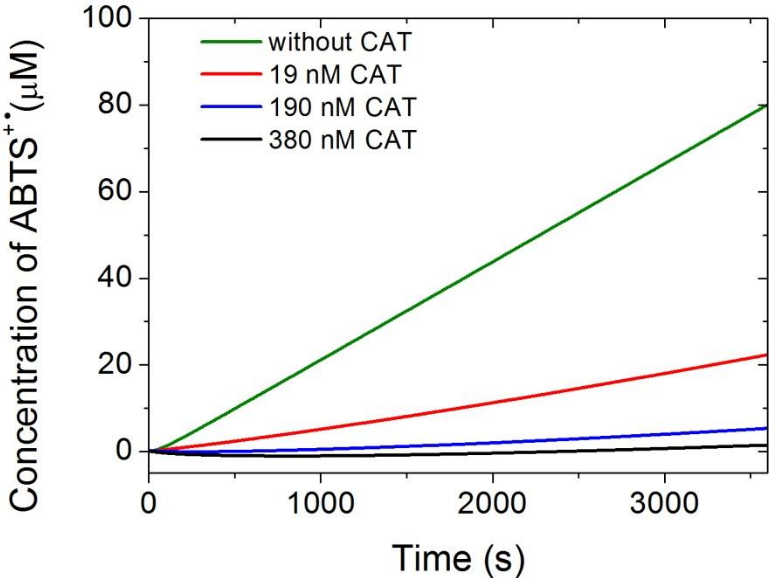

Supplementary Figure 15. Free GOx/HRP cascade with catalase competition.

Reaction Kinetics Curve Fitting. Product formation of the GOx-HRP-Catalase enzyme reactions was modeled using the following Supplementary Equations (8) and (9):

$$\frac{d[I]}{dt} = r_1 - f_{dc}r_1 - \left(\frac{V_{m,HRP}[I]}{K_{m,HRP} + [I]}\right) - \left(\frac{V_{m,Cat}[I]}{K_{m,Cat} + [I]}\right) \quad (8)$$

$$\frac{d[P]}{dt} = 2f_{dc}r_1 + 2\left(\frac{V_{m,HRP}[I]}{K_{m,HRP} + [I]}\right) \quad (9)$$

In the above equations, *[I]* denotes intermediate concentration, *[P]* denotes the product concentration, r_1 denotes the rate of the GOx reaction, V_m denotes the maximum velocity of reaction for the respective enzyme, and K_m denotes the Michaelis constant of the respective enzyme. All kinetic parameters were determined beforehand. The reaction was assumed to begin with zero initial intermediate and product concentration. As a result, the fitting parameters of the model were a time offset, which would allow adjustment for the few seconds between reaction commencement and spectrophotometer measurement, and f_{dc}.

Curve fitting of GOx-HRP-Catalase data was accomplished using MATLAB software's *lsqcurvefit* function, which performs a least squares approximation between a model function and fitting data, with bounds on fitting parameters. Direct channeling fraction was bound to the interval [0,1]. ODE integration was performed using MATLAB software's *ode45* function. The significance of proximity channeling was tested using the MATLAB lsqcurvefit function to attain a fraction of direction channeling equal to 0, with a 95% confidence interval of

(-0.0045,0.0045).

Influence of TEMg buffer. A concern is that the enzymatic activity may be different in phosphate buffer and TEMg buffer (20 mM Tris, 2 mM EDTA, 12.5 mM $MgCl_2$, pH=7.5), which is usually used to dissolve DNA origami samples. As shown in **Supplementary Figure 16**, the activities of GOx and HRP do not significantly change in TEMg buffer.

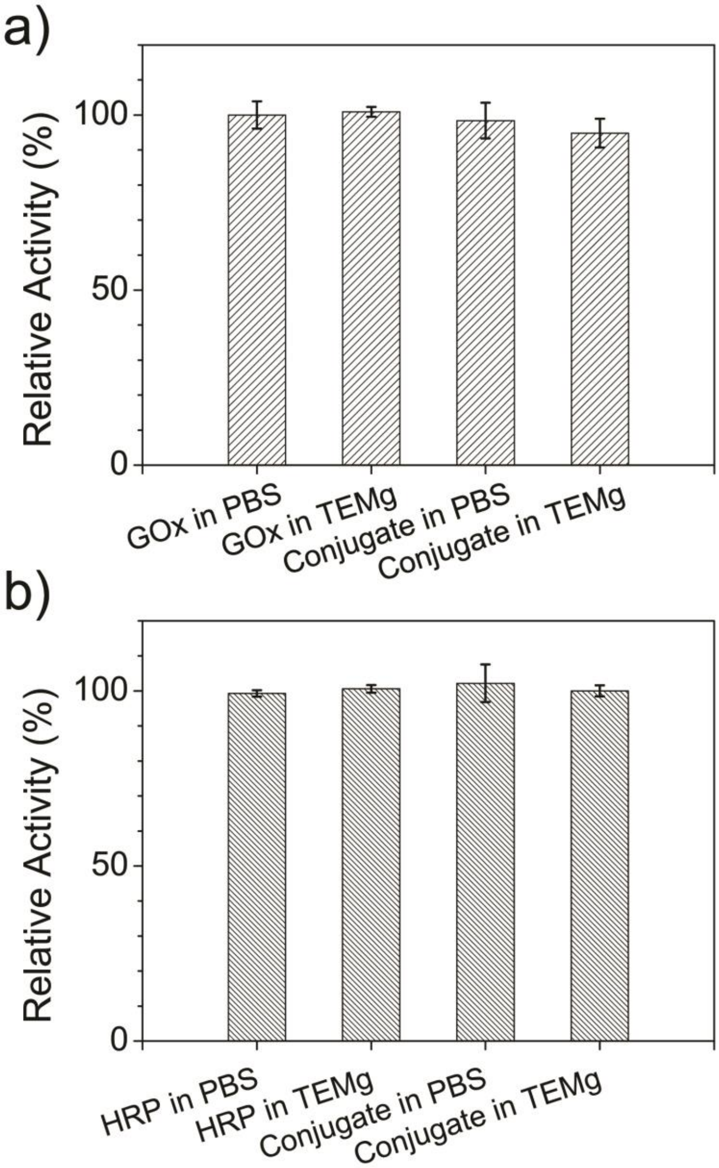

Supplementary Figure 16. Relative activities of (a) GOx and conjugated GOx in PBS and TEMg buffer; (b) HRP and conjugated HRP in in PBS and TEMg buffer. Error bars represent the standard deviations calculated from three replicates.

DNA Origami Potential Profile. The pH profile near the DNA Origami plate was modeled

using a simple Poisson-Boltzmann model. The plate was assumed to behave as an infinite sheet with electron charge density 36 nm^{-2}. This corresponds to a charge density of 5.76 C m^{-2}. The surface potential was deduced by solving Grahame's Equation:

$$\sigma = \sqrt{8C_0 \varepsilon RT} \sinh\left(\frac{F\Psi_0}{2RT}\right) \qquad (10)$$

In the above equation, σ is the surface charge density, R is the universal gas constant, T is the temperature, C_0 is the bulk salt concentration, ε is the product of the vacuum permittivity constant and dielectric constant of water, F is the Faraday constant, and Ψ_0 is the surface potential.

The potential profile near the DNA Origami surface was solved for using the Poisson-Boltzmann Equation:

$$\nabla^2\Psi = -\frac{2FC_0}{\varepsilon}\sinh\left(-\frac{F\Psi}{RT}\right) \qquad (11)$$

Since we model our system as an infinite sheet, we may invoke symmetry, and the above equation reduces to a one dimensional partial differential equation with boundary conditions:

$$\frac{\mathrm{d}^2\Psi}{\mathrm{dx}^2} = -\frac{2FC_0}{\varepsilon}\sinh\left(-\frac{F\Psi}{RT}\right) \qquad (12)$$

$$\Psi(x = 0) = \Psi_0$$

$$\Psi(x = \infty) = 0$$

This equation may be solved for analytically:

$$\Psi(x) = \frac{2RT}{F} \cdot \log\left(\frac{1 + \ exp(-\kappa x)\, tanh(\alpha)}{1 - exp(-\kappa x)\, tanh(\alpha)}\right) \qquad (13)$$

$$\alpha = \frac{F\Psi_0}{4RT}$$

$$\kappa = \sqrt{\frac{2F^2C_0}{\varepsilon RT}}$$

The pH profile is then determined by using the Supplementary Equation (14):

$$\mathrm{pH(x)} = \mathrm{pH}_b + \frac{F\Psi(\mathrm{x})}{2.3RT} \qquad (14)$$

pH dependences of GOx-HRP conjugate. Here, the final activities in different pH are limited by the GOx, in accordance with the pH-activity profile shown in Figure 6c.

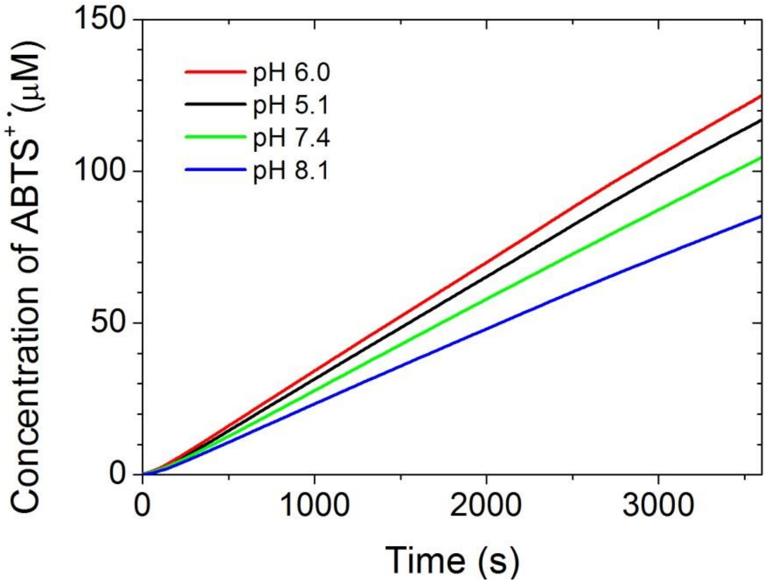

Supplementary Figure 17. Time course curves of $ABTS^{+\bullet}$ production by the GOx-HRP conjugate (1 nM) in different pH conditions and at 1 mM glucose concentration.

Supplementary References:

1. Fu, J., Liu, M., Liu, Y., Woodbury, N. W. & Yan, H. Interenzyme substrate diffusion for an enzyme cascade organized on spatially addressable DNA nanostructures. *J. Am. Chem. Soc.* **134**, 5516– 5519 (2012).

2. Zhao, Z., Fu, J., Dhakal, S., Johnson-Buck, A., Liu, M., Zhang, T., Woodbury, N. W., Liu, Y., Walter, N. G. & Yan, H. Nanocaged enzymes with enhanced catalytic activity and increased stability against protease digestion. *Nat. Commun.* **7**, 10619 (2016).
